# The impact of personality factors on delay in seeking treatment of acute myocardial infarction

**DOI:** 10.1186/1471-2261-11-21

**Published:** 2011-05-19

**Authors:** Mona Schlyter, Lena André-Petersson, Gunnar Engström, Patrik Tydén, Margareta Östman

**Affiliations:** 1 Department of Cardiology, Skåne University Hospital, 20502 Malmö, Sweden; 2 Cardiovascular Epidemiology Research Group, Department of Clinical Sciences, Lund University, 20502 Malmö, Sweden; 3 Faculty of Health and Society, Malmö University, 20506 Malmö, Sweden

## Abstract

**Background:**

Early hospital arrival and rapid intervention for acute myocardial infarction is essential for a successful outcome. Several studies have been unable to identify explanatory factors that slowed decision time. The present study examines whether personality, psychosocial factors, and coping strategies might explain differences in time delay from onset of symptoms of acute myocardial infarction to arrival at a hospital emergency room.

**Methods:**

Questionnaires on coping strategies, personality dimensions, and depression were completed by 323 patients ages 26 to 70 who had suffered an acute myocardial infarction. Tests measuring stress adaptation were completed by 180 of them. The patients were then categorised into three groups, based on time from onset of symptoms until arrival at hospital, and compared using logistic regression analysis and general linear models.

**Results:**

No correlation could be established between personality factors (i.e., extraversion, neuroticism, openness, agreeableness, conscientiousness) or depressive symptoms and time between onset of symptoms and arrival at hospital. Nor was there any significant relationship between self-reported patient coping strategies and time delay.

**Conclusions:**

We found no significant relationship between personality factors, coping strategies, or depression and time delays in seeking hospital after an acute myocardial infraction.

## Background

Little is known about the impact of psychosocial and personality factors on delays from the onset of symptoms of acute myocardial infarction (AMI) to hospital admission. O'Carroll and colleagues have found several elements that might explain failure to seek immediate medical care. Those patients who had longer time delays scored lower on neuroticism and higher on denial [1]. Different coping strategies, such as praying, waiting for symptoms to abate, resting, or self-medicating with non-prescription drugs are factors that may account for prolonged intervals of waiting before seeking medical care [2,3]. A recent study in the US found that low income levels and neighbourhoods characterized by poor social ties also contributed to such delays in seeking hospital care [4]. Difficulty in describing symptoms and chronic illness resulting from other diseases tended to confuse symptoms more in women than in men [5].

Psychosocial factors associated with adverse cardiac events can be divided into emotional factors and chronic stressors [6]. The most prominent emotional factor linked to coronary heart disease (CHD) in several studies has been depression [7,8]. Certain personality traits have specifically been linked to CHD, namely, those associated with Type D personality (characterized by distress and suppression of negative emotions) [9]. The distinctive personality traits of anxiety [10] and hostility [11] have also shown associations with CHD. Psychosocial factors that can be considered chronic stressors include low socioeconomic status, poor social support, and various situations in which individuals are exposed to stress [6]. Unique behavioural factors associated with stressful events have also been assumed to be risk factors linked to myocardial infarction (MI) and increased risk of death after such an event [12,13].

Studies have also associated prehospital delay with failure to trust others [14], problems in recognizing symptoms [15], and having insufficient (or no) insurance [16]. However, it remains vital to identify factors which can help to understand patient behaviour in this regard, since time to reperfusion is significant in determining the size of an infarction [17]; and future prognosis can depend on the interval between onset of symptoms and treatment [3]. The difficulty of making this known to the public is illustrated by the fact that extensive education campaigns in the US have failed to reduce the time in seeking acute care [18,19].

Rapid medical intervention of AMI can prevent mortality and reduce morbidity [20]. Although early arrival at hospital is necessary for successful treatment and minimalization of negative consequences, many patients fail to recognize the seriousness of their symptoms and thus unnecessarily delay getting medical attention [3,21].

The purpose of this study is to analyse the correlation of personality and psychosocial factors, with the time lag between the onset of coronary symptoms and seeking emergency hospital care.

### Methods

#### Participants and Study design

Patients admitted to the coronary care unit (CCU) at Malmö University Hospital in Sweden from July 2002 to January 2005 were eligible for the study. The CCU has a catchment area of approximately 275,000 inhabitants.

This investigation is based on the SECAMI (Secondary Prevention and Compliance following Acute Myocardial Infarction) study that explored psychological and personality factors in patients with MI and their effect on prognosis, their adherence to secondary prevention measures, and the treatment provided to them.

The sample of patients was chosen to represent an average population admitted to the CCU with a diagnosis of MI (ICD - code I21).

The inclusion criteria for this study were admission to the CCU with 1) acute MI, as diagnose by the definition of the European Society of Cardiology guidelines [22], 2) age between 18 and 70, 3) residence within the hospital catchment area, 4) adequate communication skills and knowledge of the Swedish language, and 5) the availability of research staff.

Patients were enrolled in the study within 24 hours of admission to the CCU. Clinically stable patients were informed about the study in oral and written form, and informed consent was obtained. The research nurse (MS) interviewed the patients.

A clinical psychologist performed tests measuring stress adaptation, coping strategies, personality, and depression within 36 hours of arrival at the CCU. Upon discharge from the hospital, each patient in the study was followed-up in a programme, conducted by a nurse that included lifestyle changes at the Secondary Prevention Unit at the Malmö University Hospital. The nurse who had interviewed the patients at the CCU was not involved in the secondary prevention program.

Eight hundred and forty-seven patients were eligible for the study. A total of 208 patients could not be included because they were admitted at times when the research staff was not available. An additional 101 patients were excluded due to their residence outside the hospital catchment area. Another 72 patients did not understand Swedish, and 21 more were either severely ill or judged to have insufficient mental or physical capacity to participate. Of the total population of 445 patients who fulfilled the entry criteria, 45 declined to take part in the study, leaving a pool of 400 potention interviewees. The present cohort consists of 323 patients for whom complete information on depression, personality factors, and coping strategies was available. Information about stress adaptation could be determined for a subgroup of 180 patients.

The study was approved by the Ethics Research Committee, Faculty of Medicine, University of Lund (LU230-02).

### Data Collection

#### Social and demographic data

A comprehensive questionnaire was designed in order to obtain information about risk factors for CHD. The questionnaire concerned prior illnesses, medication, occupation, educational level, marital status, nationality, smoking or snuff and alcohol consumption, as well as time from the onset of symptoms to arrival at hospital. Wherever possible, the time of onset was also checked with relatives or bystanders. The time of arrival was taken from hospital records. No data was missing. Marital status was classified as single, widowed, divorced, or married/cohabiting. Respondents could indicate their educational level as university education, intermediate level, or less than 9 years of schooling. Patients were also asked whether they were working, had retired, or were unemployed due to illness. Smoking and snuff habits were categorized as current, former, or never. Body mass index (BMI) was calculated as weight (kg)/height (m²). Personality factors and social network were assessed through administration of a Color Word Test, a unstructured coping interview, and the Beck Depression Inventory.

### Measurements of psychosocial factors

#### The serial Color Word Test (CWT)

The serial CWT a semi-experimental approach to measuring how individuals adapt to a stressful situation [23]. It is a version of the Stroop test that was originally designed to study interference and has been described in several earlier studies [12,23,24]. Words are printed in an incongruent color and the subject is asked to name the color of the print while disregarding the written word. The amount of time it takes for a subject to finish two rows of color words is measured in seconds. By presenting the test in a serial manner, it is possible to study cognitive adaptation as a process [25]. Behaviour is categorized according to emergence of time patterns that are assumed to show how individuals adapt to cognitive conflicts in everyday life [24]. André-Petersson and collaborators have shown that both the variability and the regression dimensions of the CWT are related to cardiovascular disease [13,25-27]. In each dimension, four patterns, named in accordance with their graphic appearance, can be distinguished: Stabilized, Cumulative, Dissociative, and Cumulative-Dissociative. The Dissociative and the Cumulative-Dissociative patterns are linked to difficulties managing stressful situations.

#### Beck Depression Inventory (BDI)

The BDI is a 21-item self-rating instrument used to assess the existence and severity of depressive symptoms. A cut-off threshold at established 10 points or above was used to indicate depressive symptoms in our study. The BDI has been repeatedly validated [28] and is often used to assess cardiovascular populations [29,30].

#### Coping

Coping can be described as actions or thoughts that individuals employ when dealing with stressful circumstances [31]. An unstructured interview regarding coping strategies when confronted by critical life events was included in the psychological examination. Answers were classified according to10 categories: confrontive coping, distancing, self-controlling, seeking social support, accepting responsibility, escape/avoidance, systematic problem solving, and positive reappraisal. We added two more categories: altruism and "failure to find a coping strategy". An alternative category "flexibility in coping", was also employed. Those individuals referring to more than one coping strategy were categorized as having a flexible coping style.

#### Personality

To assess the personality of participants a short version of the NEO Personality Inventory (NEO PI) was used measure neuroticism, extraversion, openness, agreeableness, and conscientiousness [32].

### Statistical analysis

Pearson's chi-square test (for dichotomous variables) and logistic regression analysis (for ordinal variables) were employed to compare background factors and clinical characteristics of men and women, using sex as a dependent variable in the calculations. The patient cohort was divided into three groups based on the time interval from onset of symptoms until arrival at hospital. For Group 1 the time was 0 to 119 minutes, for Group 2 it was 120 minutes to 6 hours, and for Group 3 it was 6 to 72 hours. Logistic regression was used to compare dichotomous variables by time delay groups. Time delay was then fitted as an ordinal variable in the logistic regression model and *p *-values for the trend over the groups was used. The logistic regression model was also used to adjust the *p *-values for age and sex. For continuous variables, a general linear model was used. The groups of time delay were used as a fixed factor and the *p *-value for the linear association over the factor levels was used. The general linear model was also used to adjust for age and sex. Finally, a stepwise multiple logistic regression, with time delays above or below 6 hours was applied as a dependent variable to assess factors independently associated with long time delays. The *p- *value for removal from the stepwise model was 0.10 in this analysis.

### Results

#### Baseline characteristics

Baseline characteristics of the patients in the study are presented in Table 1. Their mean age was 60.6 years (range 37 to 70 SD ± 8.0) for the women, and 57.7 years (range 26 to 70 SD ± 8.4) for the men. Men were more often employed (50.8%) than women (36.0%) (*p *= 0.02). A greater proportion of women lived alone, and had higher BMI than men. Depressive symptoms were more prevalent in women than in men (mean 10.5 ± 8.0 vs.7.6 ± 7.0, *p *< 0.001). Of the men, 31.7%, and 32.6% of the women had ST-elevation MI. Left ventricular ejection fraction < 40 was found in 24.0% of the men and in 27.9% of the women.

**Table 1:** Background factors and clinical characteristics of the study population

	Men		Women		
	n = 237	73.4%	n = 86	26.6%	*p *-value
Age					
26-50 years	45	19.0	10	11.6	
51-60 years	93	39.2	30	34.9	
61-70 years	99	41.8	46	53.5	0.04
Living alone	63	66.5	36	40.4	0.06
Employed or professionally active	121	50.8	32	36.0	0.02
Immigrant	46	19.4	10	11.6	0.09
Education					
Low (< 9 years)	71	30,0	26	30.2	
Intermediate	135	57.0	49	57.0	
High (University)	31	13.1	11	12.8	0.94
Medical history					
Current smoker	103	43.5	46	53.5	0.11
Previous myocardial infarction	39	16.5	12	14.0	0.59
Treatment for hypertension	90	38.0	41	47.7	0.12
Treatment for hypercholesterolemia	57	24.1	16	18.6	0.30
Diabetes mellitus	37	15.6	14	16.3	0.89
Body Mass Index ≥ 30	54	22.8	32	37.2	0.01
History of family MI	81	34.2	22	25.6	0.14

Data given as n%

MI = myocardial infarction

#### Time delay

The relationship between patient characteristics and time to hospital is shown in Table 2. Patients who had previously experienced an MI did not arrive at the hospital any earlier than patients without a prior MI, nor did age show any association with time delay. Furthermore, there was no significant relationship between scores on the neuroticism, extraversion, agreeableness, openness, or conscientiousness scale and time delay. We also found no correlation between time delay and degree of education, immigrant status, depression, or other psychosocial factors. The relationship between time delay and personality factors or depression remained insignificant after adjustment for age and sex.

**Table 2:** Distribution of clinical, psychosocial, and personality background characteristics by time to arrival at hospital

Time to hospital arrival in minutes	Group 1 0-119	Group 2 120-360	Group 3 361-4320	*p *-value	Adjusted*
n = 323	159	60	104		
Age (years)	57.7 ± 8.9	59.8 ± 7.6	58.6 ± 7.6	0.22	0.43
Men	76.7	71.7	69.2	0.17	0.22
Immigrant	18.2	16.7	16.3	0.68	0.92
Married	74.8	58.3	70.2	0.31	0.40
Education high (university)	13.8	18.3	8.7	0.28	0.29
Previous MI	15.1	13.3	15.4	0.94	0.95
STEMI	37.1	35.0	34.6	0.67	0.71
BMI ≥ 30	24.5	25.0	30.8	0.28	0.32
Current smoker	44.0	43.3	50.0	0.42	0.33
					
Beck Depression Inventory	7.8 ± 7.4	7.8 ± 6.1	9.5 ± 8.0	0.09	0.08
NEO Personality Inventory					
neuroticism	16.9 ± 9.0	17.0 ± 9.5	18.3 ± 9.2	0.26	0.25
extraversion	29.5 ± 6.8	29.2 ± 6.8	29.0 ± 6.6	0.60	0.62
agreeableness	30.5 ± 5.5	30.2 ± 5.5	29.8 ± 5.3	0.37	0.25
openness	27.7 ± 6.6	27.8 ± 6.9	26.8 ± 6.7	0.31	0.28
conscientiousness	34.5 ± 6.2	34.9 ± 6.2	33.9 ± 6.3	0.43	0.29
Coping strategies					
confrontive	12.6	8.3	8.7	0.29	0.30
distancing	17.6	11.7	12.5	0.23	0.33
self-controlling	11.3	15.0	14.4	0.44	0.51
seeking social support	8.2	20.0	8.7	0.71	0.86
accepting responsibility	9.4	8.3	9.6	0.98	0.93
escape/avoidance	8.8	10.0	10.6	0.63	0.56
systematic problem solving	17.6	10.0	16.3	0.69	0.79
positive reappraisal	1.9	1.7	2.9	0.61	0.64
no coping strategy	8.2	11.7	12.5	0.25	0.32
altruism	4.4	3.3	3.8	0.80	0.78
used more than one coping strategy	31.4	21.7	26.9	0.37	0.30

Values are mean ± standard deviation or proportions,%

*p = trend over time, adjusted for age and sex

STEMI = ST-elevation myocardial infarction

NEO = Neuroticism/Extraversion/Openness

In a logistic regression model adjusted for age and sex, there was no significant relationship between results from the CWT and a time delay of more than 6 hours, either regarding the variability or the regression dimensions in the subgroup of 180 individuals with CWT data (not shown).

A backward stepwise logistic regression analysis was performed with time > 6 hours as a dependent variable. All variables in Table 2 were entered in the first step. Only the BDI assessment was retained in the last step (Odds Ratio per 1 point increase = 1.03, 95% Confidence Interval 0.999 - 1.06, *p *= 0.06).

## Discussion

The main finding of this study is that a number of personality factors (e.g., neuroticism and agreeableness), depressive symptoms, and living alone do not seem to influence delay time in seeking acute medical care. Even though the relationship between depression and time delay neared statistical significance, we could not replicate earlier research on depression and delayed presentation after AMI [30].

In our study, neither were any of the personality factors assessed, nor depression, significantly associated with delay time. In a prospective cohort study, participants were followed for 21 years in order to determine whether mortality was influenced by neuroticism and extraversion [33]. Elevated neuroticism was found to be significantly associated with an excess risk of mortality from cardiovascular disease.

A review by Wulsin and Singal found that depressive symptoms were associated with a significantly increased risk for cardiovascular disease [7]. Psychosocial stress has been related to risk of ischemic heart disease [34]. The reasons for the correlation between psychosocial factors and cardiovascular disease are still unclear. However, our results suggest that the increased cardiovascular risk in individuals with these factors cannot be explained by less willingness to seek emergency care at the time of an acute coronary event.

The analysis of the CWT in relation to time delay was limited by a relatively small data sample, since only 180 individuals took this test. It is an exhaustive assessment procedure, and that may have reduced the number of those who participated in it. The study entitled of "Men born in 1914" showed an almost three-fold increased risk of MI in hypertensive men who had difficulties managing the stressful CWT [12].

The inventories used in the present analysis represent established concepts of personality and psychosocial stress and have shown relationships with various health conditions in several previous studies [12,35]. Research using the BDI as well as the CWT and the personality scales have shown associations with cardiovascular disease and mortality. Parakh et al. found that depression after an MI was associated with increased mortality in a short-time perspective [36]. A study using the CWT has shown that male patients who have difficulties in adapting to a stressful situation appear to have an increased risk of death following a myocardial infarction [13].

There are many instruments for measuring coping strategies, making it difficult to compare coping results between studies. We failed to find a relationship between coping and delay time in seeking treatment. Other studies have reported longer delay times associated with specific coping strategies, such as attempts to wish away symptoms, or pray that they will disappear, or trying to relax [2]. In a meta-synthesis, Lefler and Bondy [15] found several reasons that women delayed in seeking treatment for MI. The most significant were atypical presentation of symptoms, severity of symptoms, and the presence of other chronic illnesses. However, although the instruments in the present study have been used in previous investigations of cardiovascular disease, it must be acknowledged that they may be suboptimal for the purpose of studying time delay, and therefore may have failed to detect significant relationships.

We only took into account survivors after a myocardial infarction. About one-third of all persons with an acute MI die without reaching a hospital [37]. Since depression and personality factors such as neuroticism and being single have previously been associated with reduced survival [33,38], it is possible that the selective mortality of unmarried subjects or of persons with specific personality traits reduce the likelihood of any correlation and time delay in seeking hospital care. Other unmeasured factors may perhaps also explain the reasons behind the variation in time delay. For instance, patients might not consider their pain symptomatic of a dangerous condition.

Time delay may be influenced by a patient's individual decisions [21], available transportation, and geographical distance. Although the university hospital in our study is the only facility for acute somatic care in the area, it can be reached within 15 minutes from any point in the city. Ambulance transportation and acute care are provided at nominal cost. Thus, geographical distance and accessibility issues alone cannot account for the absence of a significant relationship between personality traits and time delay. In a US study, patients with no insurance or with insufficient coverage went for a considerable length of time between the onset of symptoms and seeking emergency care [16].

The present study has limitations as well as strengths. One strength is that measurements and interviews were performed by the same specialist researchers. A limitation of the study is that not all patients could be included because research personnel were not always available to interview them when they entered hospital as emergency admissions. Another shortcoming is the absence of participants older than age 70, a group which frequently only seeks acute care after some delay [3]. Our results should, therefore, not be generalized to all patients with AMI; further studies are needed to fill this gap. Another limitation involves the exclusion of patients who did not speak or read Swedish. Finally, the choice of an instrument to measure stress may have affected the participation rate in one part of the study, since some patients may have wished to avoid a test they feared being unable to handle.

## Conclusion

Although receiving acute treatment without delay is of vital importance after myocardial infarction, not much is known about any correlation between pre-hospital delay and personality factors. The present study showed no significant relationship between personality factors and depressive symptoms that would account for a time delay from the onset of MI symptoms to arrival at hospital. Nor were coping strategies or educational levels predictive of the interval before an individual would present for emergency treatment, suggesting that more research in this area is needed.

## Competing interests

Professor Gunnar Engström is employed as senior epidemiologist at AstraZeneca R&D. The other authors have no conflicts of interest to disclose.

## Authors' contributions

MS, LA-P, and PT examined and interviewed the patients. All authors contributed to the design of the study. MS and LA-P performed the statistical analysis. MS drafted the orginal manuscript and all authors provided critical input. All authors have read and approved the final version of the manuscript.

## Pre-publication history

The pre-publication history for this paper can be accessed here:

http://www.biomedcentral.com/1471-2261/11/21/prepub
